# Association Between User Engagement of a Mobile Health App for Gout and Improvements in Self-Care Behaviors: Randomized Controlled Trial

**DOI:** 10.2196/15021

**Published:** 2019-08-13

**Authors:** Anna Serlachius, Kiralee Schache, Anel Kieser, Bruce Arroll, Keith Petrie, Nicola Dalbeth

**Affiliations:** 1 Department of Psychological Medicine Faculty of Medical and Health Sciences University of Auckland Auckland New Zealand; 2 General Practice and Primary Healthcare, Faculty of Medical and Health Sciences University of Auckland Auckland New Zealand; 3 Bone and Joint Research Group, Department of Medicine, Faculty of Medical and Health Sciences University of Auckland Auckland New Zealand

**Keywords:** mobile apps, mHealth, gout, chronic disease, user engagement, illness perceptions

## Abstract

**Background:**

Mobile health (mHealth) apps represent a promising approach for improving health outcomes in patients with chronic illness, but surprisingly few mHealth interventions have investigated the association between user engagement and health outcomes. We aimed to examine the efficacy of a recommended, commercially available gout self-management app for improving self-care behaviors and to assess self-reported user engagement of the app in a sample of adults with gout.

**Objective:**

Our objective was to examine differences in self-reported user engagement between a recommended gout app (treatment group) and a dietary app (active control group) over 2 weeks as well as to examine any differences in self-care behaviors and illness perceptions.

**Methods:**

Seventy-two adults with gout were recruited from the community and three primary and secondary clinics. Participants were randomized to use either Gout Central (n=36), a self-management app, or the Dietary Approaches to Stop Hypertension Diet Plan (n=36), an app based on a diet developed for hypertension, for 2 weeks. The user version of the Mobile Application Rating Scale (uMARS, scale: 1 to 5) was used after the 2 weeks to assess self-reported user engagement, which included an open-ended question. Participants also completed a self-report questionnaire on self-care behaviors (scale: 1-5 for medication adherence and diet and 0-7 for exercise) and illness perceptions (scale: 0-10) at baseline and after the 2-week trial. Independent samples t tests and analysis of covariance were used to examine differences between groups at baseline and postintervention.

**Results:**

Participants rated the gout app as more engaging (mean difference –0.58, 95% CI –0.96 to –0.21) and more informative (mean difference –0.34, 95% CI –0.67 to –0.01) than the dietary app at the 2-week follow-up. The gout app group also reported a higher awareness of the importance of gout (mean difference –0.64, 95% CI –1.27 to –0.003) and higher knowledge/understanding of gout (mean difference –0.70, 95% CI –1.30 to –0.09) than the diet app group at follow-up. There were no significant differences in self-care behaviors between the two groups postintervention. The gout app group also demonstrated stronger negative beliefs regarding the impact of gout (mean difference –2.43, 95% CI –3.68 to –1.18), stronger beliefs regarding the severity of symptoms (mean difference –1.97, 95% CI –3.12 to –0.82), and a stronger emotional response to gout (mean difference –2.38, 95% CI –3.85 to –0.90) at follow-up. Participant feedback highlighted the importance of tracking health-related information, customizing to the target group/individual, providing more interactive features, and simplifying information.

**Conclusions:**

Participants found the commercially available gout app more engaging. However, these findings did not translate into differences in self-care behaviors. The gout app group also demonstrated stronger negative illness perceptions at the follow-up. Overall, these findings suggest that the development of gout apps would benefit from a user-centered approach with a focus on daily, long-term self-care behaviors as well as modifying illness beliefs.

**Trial Registration:**

Australian New Zealand Clinical Trials Registry ACTRN12617001052325; https://anzctr.org.au/Trial/Registration/TrialReview.aspx?id=373217.

## Introduction

The potential to harness mobile technology for improving self-management in chronic disease is substantial, as reflected by the increase in the use of mobile health and medical apps (apps to promote health and manage illness) and their popularity [1,2]. Health apps are currently one of the fastest growing app categories, with over 100,000 apps available for Android and iOS platforms in 2015 [3]. With increasing industry and government investment in medical and health apps [3,4] as well as growing research into their efficacy [5,6], health and medical apps represent an exciting opportunity to improve health outcomes among people with chronic health conditions.

There are surprisingly few evidence-based apps that have been developed for patients with gout [7], even though gout affects approximately 4% of US adults and its rates are increasing worldwide [8,9]. Gout is a treatable condition wherein monosodium urate crystals deposit in the joints and periarticular tissues, causing inflammation and painful flares. Aotearoa/New Zealand has one of the highest rates of gout worldwide [9]; Maori (indigenous New Zealanders) and Pacific adults are disproportionally affected, with a prevalence of more than 8% [10]. Although treatable, nonadherence to effective urate-lowering therapies (ULT) has been reported to be as high as >50% [11-13]. The reasons for nonadherence are complex [13], but previous literature suggests that gout medications are often viewed unfavorably, with a preference for nondrug solutions including dietary strategies [14]. Previous literature also suggests that illness perceptions play a role in self-management behaviors in gout [15,16].

Mobile health (mHealth) interventions represent a promising approach to reducing barriers to care and potentially improving adherence to effective treatments for gout. In 2016, Nguyen and colleagues [7] reviewed commercially available apps available on iOS for managing gout and found that only one app met the recommendations set in patient-focused gout management guidelines. In 2017, we expanded this search to include Android smartphones and found a similar result [17].

In addition to reviewing the content and quality of apps for gout, it is important to examine users’ experiences and engagement of apps. User engagement encompasses both how often and for how long people use apps as well as the user’s experience of the technology as a whole [18]. Engagement is therefore believed to be closely tied to effectiveness of the intervention [19]. It is estimated that approximately 23% of app users delete an app after its first use [20]; however, user engagement is often not reported as part of mHealth interventions [21] and surprisingly few mHealth interventions examine the association between user engagement and health outcomes [19,22].

Due to these gaps in the literature, the goal of our study was to examine the experiences of using a commercially available gout self-management app compared to a dietary app among patients with gout, with a focus on assessing differences in user engagement, self-care behaviors, and illness beliefs. Using a randomized controlled trial design, we compared the gout self-management app identified in the previous reviews of gout apps [7,17] and a dietary app based on the Dietary Approaches to Stop Hypertension (DASH) diet, as the DASH diet is associated with a lower risk of gout [23]. We predicted that the participants allocated to the gout self-management app would demonstrate higher user engagement, self-care behaviors, and more adaptive illness beliefs at the 2-week follow-up compared to the active control group.

## Methods

### Design

Participants were randomized to either Gout Central (treatment group), a commercially available self-management app for gout, or the DASH Diet Plan (active control group), a commercially available app based on a diet for hypertension. Seventy-two adults with gout were recruited in Auckland and randomly allocated to one of the app groups between August 2017 and May 2018. Ethics approval was granted by the Health and Disability Ethics Committee (HDEC) in New Zealand (reference number 17/NTA/38), and all participants provided written informed consent. The trial was prospectively registered with the Australian New Zealand Clinical Trials Registry (registration number ACTRN12617001052325).

### Participants and Randomization

Participants were recruited through posters in the community and from three primary and secondary clinics in Auckland, New Zealand. Inclusion/exclusion criteria included (1) a diagnosis of gout as defined by the 2015 ACR-EULAR Gout Classification Criteria [24], (2) age>18 years, (3) ability to complete forms in English and provide informed consent, and (4) ownership of or access to an android or IOS smartphone device capable of downloading apps. There were no restrictions regarding ethnic groups; however, a greater emphasis was placed on ensuring that Maori and Pacific peoples were recruited and retained in the study, as they are disproportionally affected by gout. The baseline assessment occurred face-to-face (using hard-copy questionnaires), and the follow-up assessments/questionnaires were either completed as hard-copy questionnaires and returned by post or completed online.

App group allocations were generated by a biostatistician at the School of Medicine, independent of the intervention delivery. No stratification was used. Randomization occurred via sealed envelopes labelled with sequential study numbers. A research assistant recruited participants and assigned participants to interventions.

### Intervention

After randomization, a research assistant helped participants download the app on their phones. The free versions of both apps were used in this study. Participants did not know which app was the intervention of interest/intervention group and which app was the control group.

#### Gout Central

The participants who were randomized to the treatment group were allocated to use the “Gout Central” app developed by the National Kidney Foundation for 2 weeks. This app includes information about gout and its causes, lifestyle tips for preventing gout flares, and treatment options and identifies common triggers that may cause flares. In addition, this app includes a series of health trackers such as the serum urate tracker and gout flare tracker in which the user can enter their details and track changes across time. Furthermore, this app allows users to enter their doctor appointments, log questions for their health care providers, log their medications and supplements used to manage gout and other conditions, and link them to online resources.

Participants in both app groups were advised to use all the functions they found helpful and were encouraged to use the app on a daily basis. They were also sent two text message reminders (two times during the 2-week trial period) to remind them to use the app on a daily basis. After completing the follow-up questionnaires, the participants were sent a voucher of NZ $50 to thank them for their time.

#### Dietary Approaches to Stop Hypertension Diet Plan

The active control group used the DASH diet plan app developed/sold by Chelin Apps (Android) and Diego Correa Bonini (iOS). This app provides information about the DASH diet eating plan, which has been shown to be effective in managing various health conditions such as gout [23]. The app educates users about the DASH diet, provides information to allow users to create a diet action plan, and informs users about what foods are beneficial and which ones should be avoided. This app also provides various recipes and meal ideas for breakfast, lunch, dinner, dessert, and snacks.

### Measures

#### Demographic Data

Demographic data including sex, age, and ethnicity were collected at baseline via self-report questionnaires. Current alcohol use (“Do you drink alcohol?” Response: yes/no), smoking status (“Do you smoke?” Response: yes/no), and physical activity (“Do you get at least 30 minutes of physical activity per day, eg, brisk walking?” Response: yes/no) were also assessed at baseline. Disease duration, frequency of gout flares, comorbidities, serum urate, serum creatinine, and current gout treatments were collected via self-report or from participants’ medical records. Previous and current app use (including use of health apps) was assessed in the baseline questionnaire. All the self-report questionnaires were administered online or as hard-copy questionnaires.

#### User Engagement

Our primary outcome measure was user engagement, measured using a modified user version of the Mobile Application Rating Scale (uMARS) [25] and administered after the 2-week trial. The original MARS was designed to allow app developers and health professionals to rate the quality of health apps, while the adapted uMARS scale was developed to allow users to rate health apps. The uMARS can be used to derive three separate scores: the objective app quality score, the subjective app quality score, and the perceived impact score. The objective app quality score is derived using four subscales: engagement, functionality, esthetics, and information quality. All items are rated on a 1-5 Likert scale, where 1 indicates that the app is inadequate in that domain and 5 indicates that the app is excellent in that domain. A total score is calculated by averaging across the four domains. In this study, only two of the subscales were used (engagement and information) to reduce participant burden. Therefore, only individual domain scores are presented for the two subscales without a total objective app quality score.

The subjective app quality score is derived from four items that examine overall user experience: “Would you recommend this app to people who might benefit from it?” “How many times do you think you would use this app in the next 12 months if it was relevant to you?” “Would you pay for the app?” and “What is your overall (star) rating of the app?” Lastly, the perceived impact score consists of six items that measure the impact of using the app on knowledge (“this app has increased my knowledge/understanding of the health behaviour”), attitude (“the app has changed my attitudes towards improving this health behaviour”), and behavior (“use of this app will increase/decrease the health behaviour”). The six items are reported as individual items and measured on a Likert scale ranging from 1 to 5 points (1=“strongly disagree,” 5=“strongly agree”). Lastly, the uMARS has an open-ended question, “Do you have any further comments about the app?”

The uMARS demonstrates good internal reliability for both the instrument overall and the individual subscales within the instrument [25]. The Cronbach alpha coefficient for the engagement subscale score was 0.80, for the information subscale score was 0.60, and for the app subjective quality score was 0.84.

In addition to the uMARS, two additional questions were included to assess how often and for how long participants used the apps: “In the last 14 days, on how many days did you use the app?” and “on the days that you used the app, approximately how many minutes did you spend using the app?”

#### Self-Care Behaviors

Adherence to gout self-management guidelines and self-care behaviors were assessed with a self-report questionnaire that covers behaviors related to gout management over the past 7 days. These items were adapted from the diabetes-specific Multidimensional Diabetes Questionnaire [26] and have been used previously to assess self-care behaviors in gout [27]. The items were individually scored and included behaviors such as medication (eg, “Over the last 7 days, how many of your prescribed number of gout pills [eg, allopurinol, probenecid, febuxostat or benzbromarone] did you take?”), rated on a 5-point Likert scale, from 1=“none of them” to 5 =“all of them”; exercise (eg, “On how many of the last 7 days did you participate in at least 20 minutes of exercise?”), rated on an 8-point Likert scale, from 0-7 days; and diet-related activities (eg, “How often did you follow your recommended diet over the last 7 days?”), rated on a 5-point Likert scale, from 1=“never” to 5=“always.”

#### Illness Perceptions

Illness perceptions were measured using a gout-specific Brief Illness Perceptions Questionnaire (B-IPQ) [28]. The 8-item B-IPQ examines the five key illness perception dimensions as well as items measuring the patient’s concern, understanding, and emotional response to illness. The eight items are measured on a 0-10 Likert scale, with higher scores indicative of stronger endorsement: consequences (how much gout affects the patient’s life), timeline (how long the patient thinks gout will continue), personal control (how much control the patient has over his or her gout), treatment control (how much the patient’s medication can control gout), identity (severity of gout symptoms), concern (how concerned the patient is about his or her gout), understanding (how well the patient feels he/she understand their gout), and emotional response (how much gout affects the patient emotionally.) The B-IPQ has satisfactory reliability and validity across a range of chronic illnesses [28].

### Power Calculation, Sample Size, and Statistical Analyses

Due to the lack of intervention studies that have examined differences in self-reported user engagement of health apps from which to estimate an effect size, we determined the sample size required to detect a medium effect size (0.6) between the two groups in user engagement (based on the uMARS). For the power calculation, we used an independent samples *t* test with 80% power and a 5% significance level, which indicated that 72 participants were required (36 in each group). To our knowledge, the only other study comparing user engagement scores (using the uMARS) across health apps used 5-6 participants per app group based on recommendations from the usability testing literature [29]. Our power calculation was based on recommendations for studies in which a standardized effect size is unknown [30].

**Figure 1:**
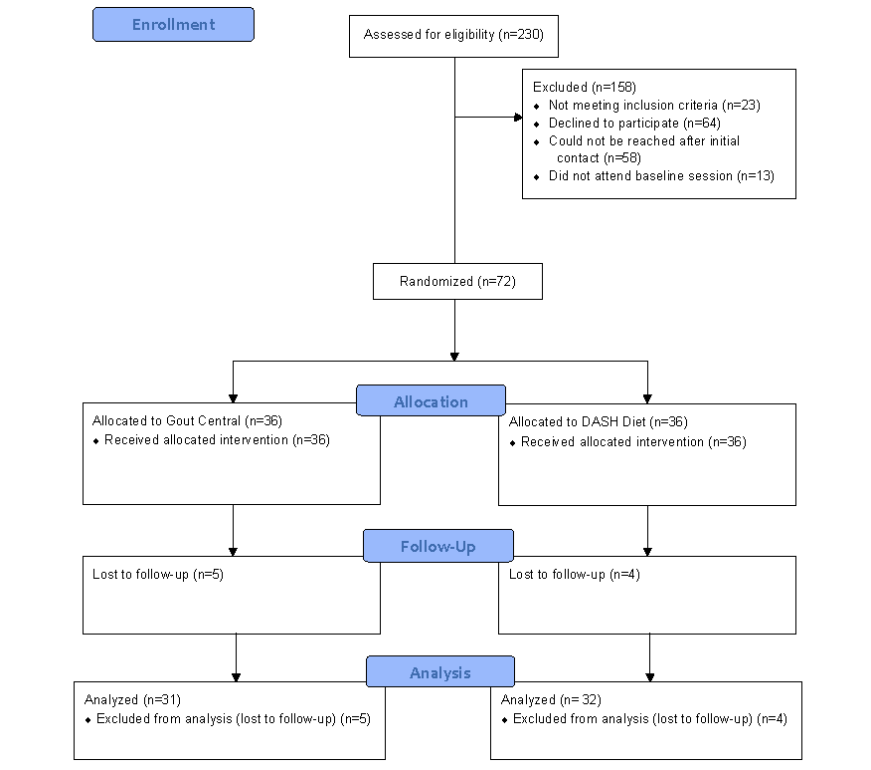
Flow diagram of participant recruitment, randomization, and attrition. DASH: Dietary Approaches to Stop Hypertension.

Of the 72 participants, 9 were lost to follow-up and did not complete any of the follow-up questionnaires (Figure 1). The analysis therefore constituted a per protocol analysis. Independent samples *t* tests and analysis of covariance (ANCOVA) for variables that were unbalanced at baseline were used to examine differences between groups postintervention in user engagement, self-care behaviors, and illness perceptions. The results remained unchanged when controlling for baseline covariates; therefore, unadjusted means are reported. Means, SDs, and 95% CIs are presented with the analyses. Effect sizes were calculated using Cohen *d*, interpreted as <0.2 (small), 0.3-0.7 (medium), and ˃0.8 (large) [31].

### Participant Feedback

Thirty-nine participants answered the open-ended question from the uMARS. The comments were analyzed independently by two researchers (AS and KS) using directed content analysis [32], appropriate where predetermined categories or concepts are being explored. After the initial stage of coding, the two researchers met to resolve any differences. The comments were initially grouped under three broad categories—positive experiences, negative experiences, and suggestions for improvement—with a frequency count for positive and negative comments. Participants’ feedback was also mapped onto the four uMARS domains: Engagement, Functionality, Esthetics, and Information.

## Results

During recruitment, 230 patients were contacted, of which 72 consented to participate and were randomized. The most common reasons for nonparticipation were not owning a smartphone or not wishing to participate in an mHealth intervention (Figure 1).

### Baseline Measures

The mean (SD) age for the total sample at baseline (n=72) was 49 (15) years, and the majority of the participants were male (86%). Sixty percent were married, 17% were in a relationship, and 23% were single. Thirty-six percent identified as European New Zealanders; 25%, as Pasifika; 19%, as Maori; and 20%, as other ethnic groups. Regarding previous app use, most participants reported using apps (95.8%), with the number of apps used ranging from 0 to 125. In addition, 40% reported already using health apps. The majority of the participants reported drinking alcohol (73.6%), but not smoking (91.7%). A total of 69% reported being physically active. Baseline demographics were similar between the treatment and control groups (Table 1), except for age, whereby the participants in the DASH app group were older.

**Table 1:** Baseline demographic and clinical characteristics (N=72).

Characteristics		Gout Central (n=36)	DASH^a^ Diet (n=36)
Age (years), mean (SD)		45 (14)	53 (15)
Sex (male), n (%)		30 (83)	32 (89)
**Ethnicity, n (%)**			
	New Zealand European	14 (39)	12 (33)
	Maori	5 (14)	9 (25)
	Pacific	10 (28)	8 (22)
	Other ethnic groups	7 (19)	7 (19)
Disease duration (years), mean (SD)		11 (13)	15 (10)
Number of flares in the past 3 months, mean (SD)		2.2 (5.1)	0.9 (1.7)
Serum urate (mmol/L) level, mean (SD)		0.41 (0.12)	0.35 (0.11)
Serum creatinine (μmol/L) level, mean (SD)		102 (46)	99 (52)
**Current gout medications, n (%)**			
	Urate-lowering therapies^b^	20 (56)	29 (81)
	Anti-inflammatory medications	16 (44)	11 (31)
	Polypharmacy (≥5 long-term medications)	4 (11)	10 (28)
**Comorbidities (five most prevalent), n (%)**			
	Hypertension	3 (8)	6 (17)
	Type 2 diabetes	3 (8)	3 (8)
	Kidney disease	4 (11)	1 (3)
	Cardiovascular disease	2 (6)	5 (14)
	Hypercholesterolemia	1 (3)	1 (3)

^a^DASH: Dietary Approaches to Stop Hypertension.

^b^Urate-lowering therapies: allopurinol and febuxostat; anti-inflammatory medications: colchicine, nonsteroidal anti-inflammatory drugs, and corticosteroids.

Regarding baseline clinical measures, the majority of the sample (68%) reported currently taking ULT, 38% reported taking anti-inflammatory medications, and an additional 19% reported being on more than five long-term medications. The mean serum urate level of the participants at baseline was 0.38 (0.11) mmol/L. In addition, 44% reported other comorbidities, with the most common reported as hypertension, type 2 diabetes, kidney disease, cardiovascular disease, and hypercholesterolemia. The clinical variables were similar between the treatment and control groups, except for the number of participants currently taking ULTs, which were higher in the control group (control: n=29 vs treatment: n=20; Table 1).

When examining illness perceptions at baseline in the two groups, the following beliefs were unbalanced: timeline, personal control, treatment control, and identity beliefs (Table 2). Self-care behaviors were similar at baseline between the two treatment arms.

**Table 2:** Mean differences in illness perceptions at baseline and follow-up.

Illness perceptions (score range 0-10)		Gout app, mean (SD)	DASH^a^ app, mean (SD)	Mean difference (95% CI)		*P* value^b^
**Consequences beliefs**					.04	
	Baseline	4.50 (3.32)	4.11 (3.51)	–0.39 (–1.99 to 1.22)		
	Follow-up	4.61 (2.63)	2.19 (2.36)	–2.43 (–3.68 to –1.18)		
**Timeline beliefs**					.70	
	Baseline	5.94 (3.54)	8.03 (2.90)	2.08 (0.56 to 3.61)		
	Follow-up	6.81 (3.31)	7.19 (3.72)	0.38 (–1.40 to 2.16)		
**Personal control beliefs**					.28	
	Baseline	5.90 (3.02)	7.67 (2.43)	1.76 (0.48 to 3.05)		
	Follow-up	6.90 (2.66)	7.56 (2.08)	0.66 (–0.54 to 1.86)		
**Treatment control beliefs**					.54	
	Baseline	7.44 (2.69)	8.92 (1.54)	1.47 (0.44 to 2.50)		
	Follow-up	7.42 (3.00)	7.88 (2.81)	0.46 (–1.01 to 1.92)		
**Identity beliefs**					.001	
	Baseline	5.11 (2.94)	3.64 (3.09)	–1.47 (–2.89 to –0.06)		
	Follow-up	4.06 (2.25)	2.09 (2.32)	–1.97 (–3.12 to –0.82)		
**Concern beliefs**					.26	
	Baseline	5.89 (3.07)	5.67 (3.43)	–0.22 (–1.75 to 1.31)		
	Follow-up	5.26 (3.01)	4.34 (3.38)	–0.91 (–2.53 to 0.70)		
**Understanding beliefs**					.23	
	Baseline	7.44 (2.26)	8.03 (1.81)	0.58 (–0.38 to 1.55)		
	Follow-up	8.13 (1.96)	7.34 (3.07)	–0.79 (–2.09 to 0.52)		
**Emotional response beliefs**					.002	
	Baseline	4.81 (3.46)	4.56 (3.83)	–0.25 (–1.97 to 1.47)		
	Follow-up	5.00 (3.01)	2.63 (2.84)	–2.38 (–3.85 to –0.90)		

^a^DASH: Dietary Approaches to Stop Hypertension.

^b^*P* value refers to analysis of covariance for between-group comparisons postintervention.

### Differences in User Engagement Between Groups Postintervention

With regard to the engagement and information subscale scores, participants rated the gout app as more engaging than the dietary app, with a mean difference of –0.58 (*P*=.003; effect size=0.77), and as more informative than the dietary app, with a mean difference of –0.34 (*P*=.04; effect size=0.53). Although the subjective app quality score (derived from four items that examine overall user experience) was also higher in the gout app group than in the dietary app, this difference was not statistically significant, with a mean difference of –0.36 (*P*=.11). Lastly, when evaluating the six individual items that examined perceived impact of the app, the only statistically significant differences were found in awareness of the importance of gout (mean difference of –0.64; *P*=.049; effect size=0.51) and knowledge/understanding of gout (mean difference of –0.70; *P*=.03; effect size=0.57), which were both higher in the gout app group than in the dietary app group. There was little evidence that either group used the app more during the 2-week trial (in terms of days used or minutes), with *P* values of .81 and .52, respectively (Table 3).

**Table 3:** Differences in user engagement between app groups postintervention measured by the user version of the Mobile Application Rating Scale (score range: 1-5).

Measures	Gout app (n=31)	DASH^a^ app (n=32)	Mean difference (95% CI)	*P* value
Engagement subscale score	3.26 (0.73)	2.68 (0.77)	–0.58 (–0.96 to –0.21)	.003
Information subscale score	3.92 (0.51)	3.58 (0.76)	–0.34 (–0.67 to –0.01)	.04
Subjective app quality score	3.06 (0.82)	2.70 (0.94)	–0.36 (–0.81 to 0.08)	.11
Perceived impact: Awareness	3.42 (1.15)	2.78 (1.36)	–0.64 (–1.27 to –0.003)	.049
Perceived impact: Knowledge/understanding	3.32 (1.14)	2.63 (1.26)	–0.70 (–1.30 to –0.09)	.03
Perceived impact: Attitudes	3.19 (1.17)	2.59 (1.46)	–0.60 (–1.27 to 0.07)	.08
Perceived impact: Intention to change	3.06 (1.21)	2.56 (1.32)	–0.50 (–1.14 to 0.14)	.12
Perceived impact: Help seeking	2.84 (1.10)	2.59 (1.29)	–0.24 (–0.85 to 0.36)	.42
Perceived impact: Behavior change	3.10 (1.19)	2.59 (1.27)	–0.50 (–1.12 to 0.12)	.11
App use (days)	7.90 (3.95)	8.13 (3.27)	0.22 (–1.60 to 2.05)	.81
App use (minutes)	11.34 (13.00)	9.64 (6.91)	–1.70 (–6.92 to 3.52)	.52

^a^DASH: Dietary Approaches to Stop Hypertension.

### Differences in Self-Care and Illness Perceptions Postintervention

Independent samples *t* tests and ANCOVA (adjusting for age, ULT, and baseline illness perceptions) were conducted to examine differences postintervention. There were no differences in any self-care behaviors between the two groups (*P*>.05) postintervention (results not tabulated). The gout app group demonstrated stronger negative beliefs regarding the impact of gout (mean difference 2.43; *P*=.04; effect size=0.97), stronger beliefs regarding the severity of symptoms (mean difference of –1.97; *P*=.001; effect size=0.86), and a stronger emotional response to gout (mean difference of –2.38; *P*=.002; effect size=0.81) at follow-up. None of the other illness beliefs demonstrated differences postintervention (Table 2).

### Participant Feedback

The comments from the participants were grouped under three broad categories: positive experiences, negative experience, and suggestions for improvement. Textbox 1 presents a summary of the qualitative feedback, including how the feedback mapped onto the four domains of the uMARS (Engagement, Functionality, Esthetics, and Information).

**Summary of participants’ positive/negative feedback and suggestions for improvement grouped according to the four domains of the user version of the Mobile Application Rating Scale (Engagement, Functionality, Esthetics, and Information).**

**Gout app**

**Engagement**

Positive experiences:

- Tracking medications and urate levels
- Setting reminders
- Relevant for people who are newly diagnosed
- Useful during flare-ups

Negative experiences:

- Not useful for ongoing self-management of gout
- Lacking novelty
- Only useful if person has regular blood tests
- Not relevant to New Zealanders and different ethnic groups
- Not adapted for people with low health literacy

Suggestions for improvement:

- Provide more information for Pacific peoples
- Provide more videos and interactive features
- Add links to healthcare team

**Functionality**

Suggestions for improvement:

- Ability to enter more data
- Ability to track foods

**Esthetics**

Suggestions for improvement:

- Include more visual content

**Information**

Suggestions for improvement:

- Graphing urate levels
- Add links to other sources of information

**Dietary Approaches to Stop Hypertension**

**Engagement**

Negative experiences:

- Not motivating
- Not relevant to New Zealanders
- Not relevant to different cultural and ethnic groups
- Not relevant to gout

Suggestions for improvement:

- Customizing/tailoring app to the individual
- Provide social support
- More interactive content

**Functionality**

Negative experiences:

- Hard to use

Suggestions for improvement:

- Make it more simple

**Esthetics**

- Busy/confusing interface

**Information**

Positive experiences:

- Good recipes

Negative experiences:

- Poor dietary advice
- Not adapted for people with lower health literacy

Suggestions for improvement:

- Tracking/logging information

#### Positive Experiences

Positive feedback from the gout app group (n=10) reflected their satisfaction with the app’s ability to track health information, set reminders, and monitor self-care behaviors.

I particularly liked being able to track medicines and set reminders; look at graphs showing urate levels; list all my meds in one place; and be reminded to drink more water.

Participant #66, Gout app

In contrast, only two respondents in the DASH app group (n=2) provided positive feedback, both based on the information and dietary content of the app.

Very good app, very informative. Great recipes.

Participant #40, DASH app

#### Negative Experiences

Feedback from the gout app group suggested that the app was better suited to people who were newly diagnosed or patients with frequent flare-ups, rather than for long-term self-management.

I have only had a gout attack 3 times in the last 10 years. I consider that not having gout attacks regularly negates the use off the app and is not much help in my case. Also it is USA of origin and some parts are not much use in NZ.

Participant #10, Gout app

Other negative feedback from both app groups (n=3 for the Gout app and n=8 for the DASH app) included the lack of customization to New Zealand or lack of tailoring to ethnic groups more prone to gout in New Zealand such as Maori or Pacific Peoples.

Should be more customisable...Have current or relevant stats applicable to Pacific People. Go to gout sites or locations for help.

Participant #7, Gout app

#### Suggestions for Improvement

Both app groups suggested the need for more interactive features such as video content and links to health professionals or other patients.

For millennial users, it would be better to put more interactive features such as a link to videos explaining gout in a visual way...it would be better to not just link the app to our personal healthcare team but to have an online chatting with other available healthcare personnel (possibly to create a series of interactive Q&A with some available healthcare personnel).

Participant #9, Gout app

Wouldn’t it be nice to have an interactive function with other users and create a network that allows people to connect and support each other.

Participant #19, DASH diet app

Other suggestions for improvement included simplifying information, especially for people with low health literacy or non-English speakers.

This app also has very useful information that is only good for those who understand the jargon used. If I have no clue about the language used I’m not going to spend time trying to decipher the information. This part of the app needs to be simplified and made fun for all people and not just an educated few.

Participant #55, DASH diet app

The biggest challenge for the use of this app would be literacy both in Health and English. People with English as a second language would struggle.

Participant #58, Gout app

## Discussion

This is the first study to examine the impact of a commercially available gout app on health outcomes. This is also the first study to examine the utility of a commercially available gout app from the patient’s perspective, by examining user engagement. Our primary hypothesis was largely confirmed by the findings, with the gout self-management app demonstrating higher user engagement scores than the dietary app. However, this did not translate to improvements in self-care behaviors. The findings regarding illness beliefs at follow-up were more mixed, with the gout self-management app associated with stronger consequence beliefs, illness identity beliefs, and emotional response beliefs than the dietary app.

Several possible reasons exist to explain why higher user engagement did not translate to improvements in self-care behaviors. First, despite higher uMARS scores, there was no difference in time that participants spent on or used to access the gout app compared with the dietary app during the study. The average number of days spent using the app (of 14 days) for both groups was only 8. Both the apps in this study provided no feedback or goal-setting functions, thus requiring the user to be intentional about their app usage and access it without personalized feedback or any specific behavior change strategies. Second, as suggested by the participant feedback, it is possible that participants determined that the gout app (Gout Central) would only be helpful during a gout flare and thus used it less. This explanation may provide insight into why there was no difference in usage time between the two apps and no changes in self-care behaviors. Even though the gout app was chosen because it was the best available app for gout [7,17], it may not provide all the appropriate tools necessary to manage gout when it is asymptomatic. Therefore, Gout Central may not meet the needs of users in terms of continuous self-management and care of the condition between flares.

A third possible explanation is that there was little integration with daily self-management behaviors in gout. Many existing health apps focus on providing educational content, basic health monitoring, or various reminders [33], but fail to fully utilize the unique capabilities of smartphone technology (eg, real-time data collection and data visualization technology) [34]. For gout, specifically, this may mean daily medication reminders, flare diaries, visualizing serum urate fluctuations, and food diary capabilities, which encourage a user to engage with their self-care regardless of whether they are having a flare. Real-time data tracking would be especially desirable for people who are testing their serum urate levels using commercially available test meters that provide an immediate result, which would then allow for real-time serum urate tracking. Even though the gout app provided a wealth of information about gout as well as some opportunities for data tracking, real-time reminders and real-time data tracking were both missing.

Our findings regarding illness perceptions were more mixed. The more engaging gout app resulted in stronger negative illness perceptions about gout at follow-up, including stronger beliefs regarding the negative impact of having gout, the severity of symptoms, and the emotional response to gout. It is possible that using the gout app reminded participants about the negative aspects of their illness or the seriousness of their illness, which, over the long-term, may positively impact self-care behaviors. For example, in a study of patients with kidney disease, having stronger illness identity beliefs (ie, beliefs regarding severity of symptoms) was associated with more proactive coping [35]. On the other hand, we have previously found that stronger consequence beliefs and emotional response beliefs are associated with increased disability and mortality in gout [15,16]. It is also possible that negative illness beliefs (ie, more pessimistic beliefs about gout) may impact the app use itself, as participants may not want to be reminded that they have gout. Due to the short follow-up in this study, we cannot confirm either of these possibilities or whether the change in illness perceptions will be sustained or influence self-care behaviors in the long-term.

Targeting illness perceptions presents another promising area for future research in mHealth interventions. Only a handful of mHealth interventions have attempted to modify illness perceptions [36-38], with promising effects on outcomes including medication adherence as well as objective health outcomes. Considering that many commercially available health apps lack theory or evidence-based behavioral strategies [39-41], this could be an important area for future research.

The open-ended participant feedback largely supported our quantitative results, with more positive comments recorded overall for the Gout Central app than the DASH diet app. The participant feedback also highlighted that gout patients want apps that allow them to track health-related information, that are customized to their needs, that provide interactive features, and that are simple to use.

This study had many strengths: It is the first study to examine the efficacy of a commercially available gout app on self-reported health outcomes and to consider the association between user engagement and health outcomes in gout. It also successfully recruited a diverse group of patients of whom 43% were Maori or Pasifika. However, there are several limitations that should be noted. As we did not develop the apps, we were also unable to objectively examine user engagement and had to rely on self-report. Furthermore, after data collection was completed for this study, the gout app Gout Central was updated; therefore, these results may not reflect the most up-to-date version of the app. Another limitation was the short follow-up duration. Despite this short time period, there was some loss to follow-up, which necessitated a per protocol analysis rather than an intention-to-treat analysis. Lastly, these findings may not be generalizable to other populations.

The findings from this short-term trial demonstrate the many challenges associated with not only developing engaging and effective apps for patients with chronic health conditions but also the challenges present in testing commercially available apps. Our findings suggest that, at least over a period of 2 weeks, using the gout app was more engaging than using a general dietary app for patients with gout, but that the gout app is unlikely to change self-care behaviors in the short-term. It is becoming increasingly clear in the mHealth literature that the design process needs to be user-centered in order to produce the best outcomes for patients [42-44]. Work in this area is currently underway by Nguyen and colleagues [45], who have recently developed a gout self-management app. User-centered design has been tested in apps for type 1 diabetes, heart health, and asthma and has shown favorable outcomes in the preliminary stages [44]. Adopting a user-centered development approach would likely result in higher-quality apps, which are useful and accessible [46].

In conclusion, working with gout patients to develop an app that best suits their needs, targets daily self-management behaviors in between flares with specific behavior change strategies, and modifies illness beliefs could be a promising way forward in the use of mHealth to manage gout.

## Appendix

Multimedia Appendix 1 CONSORT‐EHEALTH checklist (V 1.6.1).

## Abbreviations

ANCOVA: analysis of covariance
B-IPQ: Brief Illness Perceptions Questionnaire
DASH: Dietary Approaches to Stop Hypertension
HDEC: Health and Disability Ethics Committee
mHealth: mobile health
uMARS: user version of the Mobile Application Rating Scale
ULT: urate-lowering therapies
